# Buckling Force Variability of Semmes–Weinstein Monofilaments in Successive Use Determined by Manual and Automated Operation

**DOI:** 10.3390/s19040803

**Published:** 2019-02-16

**Authors:** Manabu Chikai, Shuichi Ino

**Affiliations:** Human Informatics Research Institute, National Institute of Advanced Industrial Science and Technology (AIST), Tsukuba 305-8566, Japan; s-ino@aist.go.jp

**Keywords:** diabetic peripheral neuropathy screening, Semmes–Weinstein monofilament test, buckling force, human hand motion, human factor

## Abstract

(1) Objective: This study was conducted with the objective of characterizing the variability of a force on a simulated skin surface using the Semmes–Weinstein monofilament test (SWMT). (2) Research Design and Methods: Two distinct experiments were performed to determine the effects of human hand motion variability on the monofilament buckling force, and to determine the monofilament’s mechanical properties using a positioning stage. (3) Results: In manual operation (by human hand motion), the buckling force over the ten compressions decreased by over 10%, and the human hand motion variations during the SWMT may have impacted the buckling force. When the SWMT was performed under manual control, the buckling force was closely correlated with the number of compressions. In automated operation (by positioning stage), the buckling force was affected not only by the number of compressions but also by both the velocity and the contact angle between the monofilament and the skin surface. (4) Conclusions: The buckling force decreased in ten successive compressions, independent of the hand motion. Hence, medical staff need to consider not only the operator’s hand motion but also the effect of repeated trials.

## 1. Introduction

In recent years, the International Diabetes Federation (IDF) [1] reported that 425 million people around the world suffer from diabetes, and it is estimated that 629 million people will have diabetes by 2045. Diabetic peripheral neuropathy causes hypoesthesia—loss of sensation or touch—in a patient’s toes [1,2,3,4]. Consequently, methods that can enable the early and easy detection of neuropathy and the tracking of disease progression are essential.

At present, several methods for measuring tactile sensitivity are used clinically in diabetic peripheral neuropathy screening [5]. One such method is the Semmes–Weinstein monofilament test (SWMT) [6,7], which can be conducted at the patient’s bedside due to its ease and simplicity [8,9,10]. This method is performed by a medical professional pressing the monofilament perpendicular to the surface of the skin on the patient’s foot until there is bowing of the monofilament [10,11]. The sizes of the Semmes–Weinstein monofilaments increase in an approximately logarithmic scale, in line with Weber’s law [12]. They are not only used clinically in diabetic peripheral neuropathy screening but also at the threshold of tactile sensitivity in microneurography studies [13]. Some studies [14,15] have emphasized the importance of establishing a correlation between the detection threshold and the size of the monofilaments used in diabetic peripheral neuropathy screening. Singh et al. [14] determined that the inability to perceive the 5.07/10 gf monofilament is associated with clinically significant large-fiber neuropathy. Kamei et al. [15] stated that the 4.31/2 gf monofilament is useful in clinical tests for diabetic neuropathy due to low time requirements and low cost.

However, several studies [16,17,18,19,20,21,22] have previously reported concerns about the mechanical properties of monofilaments (for a review, see [16]). For example, Booth et al. [17] reported that the maximum recovery of the buckling force in any monofilament is achieved within 24 h and discussed the variability of force on a simulated skin surface in the results of the SWMT. Massy-Westropp [18] reported that the monofilament does not repeatedly generate the same values for similar tasks, while Haulola et al. [19] reported that the monofilament is influenced by changes in the environmental temperature and humidity. Further, Lavery et al. [20] reported that the buckling force of a new monofilament is only stable for seven to nine days, or for the evaluation of 70 to 90 patients. Chikai et al. [21] compared a new, multi-use monofilament (with the same buckling force, i.e., 5.07/10 gf) with an old one, and showed that the buckling force of the monofilament decreased through numerous test trials. In contrast, Bell-Krotoski et al. [22] showed that the SWMT provides a repeatable instrument stimulus with a small standard deviation in contrast to other handheld test instruments in a variety of clinics. Further, they reported statistically significant differences for age and measuring site with small changes in a monofilament which were so sensitive that it was advisable to stay within the same kit manufacturer when testing the same participants. While these studies reported the characteristics of the buckling force mainly from a long-term perspective, the influence of the operator’s hand motion has also been investigated. For example, Chikai et al. [21] compared the human hand motion in the SWMT and reported that human hand motion with the monofilament varied according to the participants. However, the effect of the variability of the buckling force from a short-term perspective and the effect of the operator’s hand motion on the variability remain unknown.

Consequently, this study was conducted with the objective of characterizing the variability of a force on a simulated skin surface using the Semmes–Weinstein monofilament test (SWMT). Specifically, we focused on the variability of the force over the short term. To this end, we examined the variability of the buckling force in the SWMT for both manual and automated operation in of 10 successive trials. The concept underlying the new testing system being developed in our research work [23] is based on the use of tactile stimuli.

## 2. Materials and Methods

Two experiments were conducted to examine the effects of (1) hand motion on the variability of the buckling force of the monofilament and (2) the number of compressions of the monofilament on the variability of the buckling force of the monofilament during automated operation.

**Figure 1:**
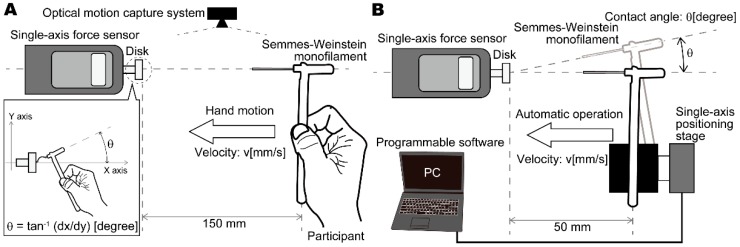
Experimental measurement apparatus: (**A**) under manual (human) operation, hand motion during monofilament application is measured using an optical 3D motion capture system, and the monofilament buckling force is measured using a single-axis force sensor; (**B**) under automated operation, the monofilament is applied using a single-axis positioning stage controlled by a stepping motor and a personal computer with programmable software.

In the first experiment, we measured the human hand motion in the monofilament test using an optical motion capture system (Natural Point Co., LTD., OptiTrack V120:Trio, Corvallis, OR, USA). We calculated and compared the velocity of each participant’s hand motion and the contact angle of the monofilament. In the second experiment, we measured the buckling force of the monofilament using an automated positioning stage with a precision motor (SIGMA KOKI Co., LTD., HPS60-20X, Tokyo, Japan). Figure 1 shows the measurement apparatus setup. In experiment 1, the force sensor was mounted on a disk substituting for the skin surface, 150 mm left of the center position of the participant’s hand (see Figure 1A). In experiment 2, the force sensor was mounted on a disk 50 mm left of the center position of the monofilament (see Figure 1B).

### 2.1. Experiment 1: Manual Operation

In this experiment, nine participants (six men and three women; mean ± SD age, 40.1 ± 10.6 years) who could operate the monofilament with their right hand, and who had no experience with medical care were recruited. The protocol was approved by the Institutional Review Board of the National Institute of Advanced Industrial Science and Technology, and all subjects provided informed consent.

A 5.07/10 gf multi-use monofilament, supplied by the manufacturer with a plastic handle, was utilized. We measured the hand motion of the participants using an optical motion capture system (Natural Point Co., LTD., OptiTrack V120:Trio, Corvallis, OR, USA), and the buckling force of the monofilament using a single-axis force sensor (NIDEC-SHIMPO CORPORATION, FGP-0.5, Kyoto, Japan). The motion capture system collected data at a rate of 120 samples per second (i.e., a 120 Hz sampling frequency) and the force sensor operated at 100 Hz sampling frequency during each performed task.

The procedure of the SWMT was outlined in a reference guide for all participants who operated the monofilament. In this experiment, an operation method was performed in which the monofilament was pressed to the disk substituting for the skin surface for approximately 1.5 s. The participants were seated at a work chair with the monofilament and the attached permanent plastic handle held in their right hand. They then pressed the monofilament against the disk and force sensor to measure the buckling force. The participants repeated each trial ten times, and the experiment was conducted twice for each participant. We calculated the velocity of the hand motion and the contact angle of the monofilament using the locus data of the 3D hand motion (see Figure 1A).

### 2.2. Experiment 2: Automated Operation

**Figure 2:**
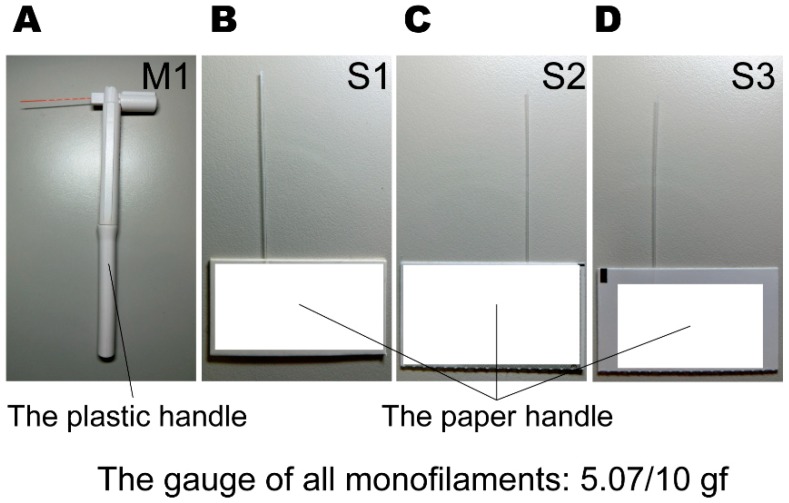
The total four 5.07/10 gf monofilaments: (**A**) the multi-use monofilament (M1); (**B**–**D**) the single-use monofilaments (S1, S2, and S3).

Four 5.07/10 gf monofilaments were used, comprising one multi-use monofilament (denoted M1) and three single-use monofilaments (denoted S1, S2, and S3) (see Figure 2). The monofilaments were each moved using a single-axis positioning stage (SIGMA KOKI Co., LTD., HPS60-20X, Tokyo, Japan) controlled by a precision stepping motor with a pulse driver unit using a PC with programmable software installed (National Instruments Co., LTD., LabVIEW, Austin, TX, USA) (see Figure 1B). The force sensor collected data at a rate of 100 samples per second (a 100 Hz sampling frequency) during each task.

The monofilaments were each pressed against the surface for 1.5 s in each of the 10 trials, as described in the reference guide (the same as in experiment 1), and the time interval between trials was 3 s. This experiment was performed twice for each of the following protocols:

(a) Velocity protocol: The velocity of the positioning stage set respectively to 1, 5, and 10 mm/s and the contact angle set to 0°.
(b) Angle protocol: The contact angle between the monofilament and the disk with the force sensor set respectively to 0, 10, and 20° and the stage speed set to 10 mm/s using the 5.07/10 gf multi-use monofilament.
(c) Different monofilament protocol: The velocity of the positioning stage set to 10 mm/s and the contact angle set to 0° using all four 5.07/10 gf monofilaments.

These protocols are described in the results of experiment 1.

### 2.3. Data Analysis

The statistical significance of the observed monofilament buckling force differences obtained following the various experimental trials was determined by one-way analysis of variance (ANOVA). For multiple comparisons of the differences between the different number of trials, the Tukey–Kramer test at levels 0.01 and 0.05 was used.

## 3. Results

### 3.1. Experiment 1: Manual Operation

**Figure 3:**
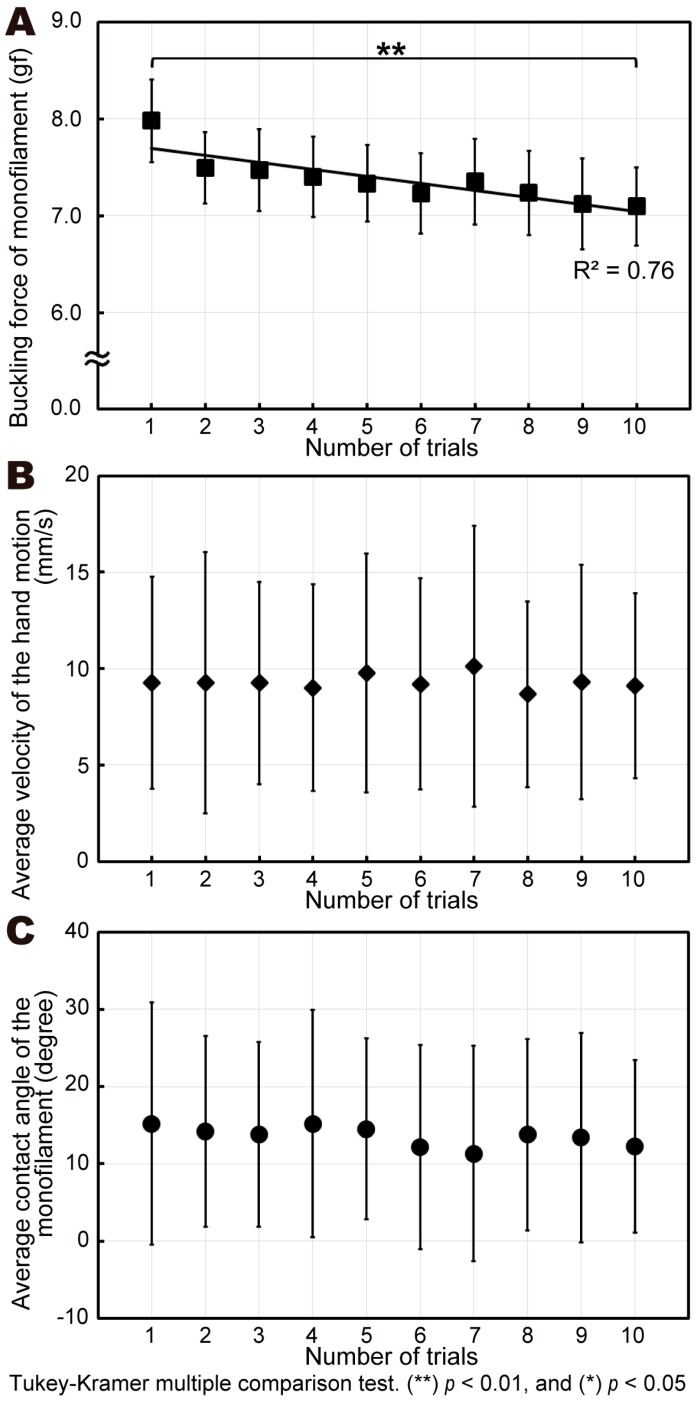
Experimental results of manual (human) operation: (**A**) monofilament buckling forces for each trial; (**B**) average velocity of human hand motion; (**C**) average contact angle of the monofilament.

We measured the human hand motion with a monofilament, simulating the SWMT used in clinical practice. As shown in Figure 3, the average buckling force of the first compression for all participants was 8.0 gf; specifically, it was 7.1 gf for the tenth compression, and the average for all 10 trials was 7.4 ± 0.5 gf. The buckling force over the 10 compressions decreased by over 10%. The correlation coefficient (*R*^2^) between the buckling force and the number of trials was 0.76, indicating that the buckling force correlates closely with the number of compressions. There were significant differences between the first and tenth trials (*p* < 0.01) (see Figure 3A), which explains why the decrease in the buckling force correlated closely with the number of compressions. The SWMT must consider that the buckling force decreased after the first compression trial. The average velocity of the hand motion and the average contact angle for all participants were 9.3 ± 5.7 mm/s and 13.6 ± 12.9 °, respectively (see Figure 3B,C). When the SWMT was performed under manual control, the buckling force was closely correlated with the number of compressions.

### 3.2. Experiment 2: Automated Operation

**Figure 4:**
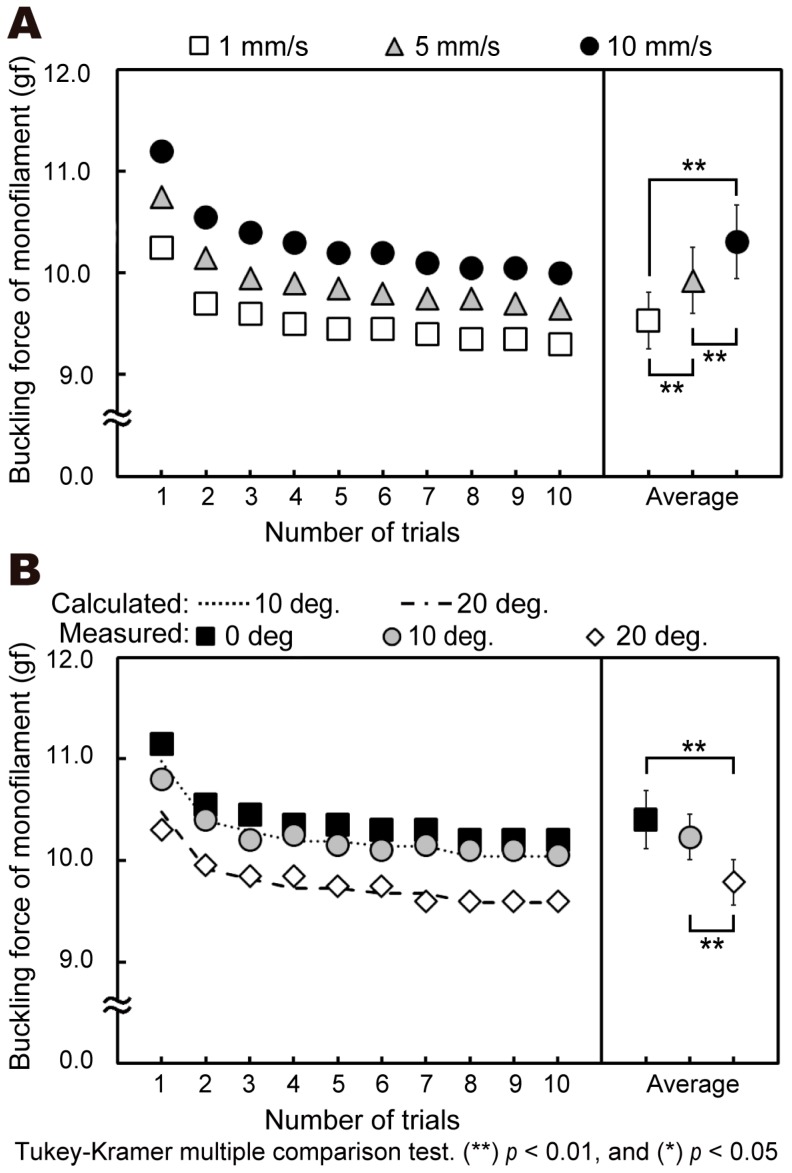
Experimental buckling force results for different operational scenarios under automated operation using the multi-use monofilament: (**A**) positioning stage velocities of 1, 5, and 10 mm/s; (**B**) monofilament contact angles of 0°, 10°, and 20°.

First, we compared the buckling force in a number of trials using the 5.07/10 gf multi-use monofilament, as shown in Figure 4. When the positioning stage velocity was 1 mm/s, the buckling force was 10.3 gf for the first trial and 9.3 gf for the tenth trial, with an average value of 9.5 ± 0.3 gf for the 10 compressions. When the velocity was 5 mm/s, the buckling force was 10.8 gf for the first trial and 9.7 gf for the tenth trial, with an average buckling force for the 10 compressions of 9.9 ± 0.3 gf. At 10 mm/s, the buckling force was 11.2 gf for the first trial and 10.1 gf for the tenth trial, with average buckling force of 10.3 ± 0.4 gf. There were significant differences between the results at the three velocity values (*p* < 0.01) (see Figure 4A), as well as between the values for the first, second, and tenth trials for each velocity. These results indicate that the buckling force is correlated with the compression velocity.

When the contact angle was 0°, the buckling force was 11.2 gf for the first trial and 10.2 gf for the tenth trial, and the average value for all 10 compressions was 10.4 ± 0.3 gf. When the contact angle was 10°, the average buckling force of the 10 compressions was 10.2 ± 0.22 gf, and at 20°, the average was 9.8 ± 0.2 gf. There were significant differences between the values at the three contact angles (0, 10, and 20°) (*p* < 0.01) (see Figure 4B). We calculated the expected buckling forces using a trigonometric function as 10.4 gf (square markers), 10.2 gf (dotted line), and 9.8 gf (dot-and-dash line), respectively. The measured forces accurately matched the calculated buckling forces.

**Figure 5:**
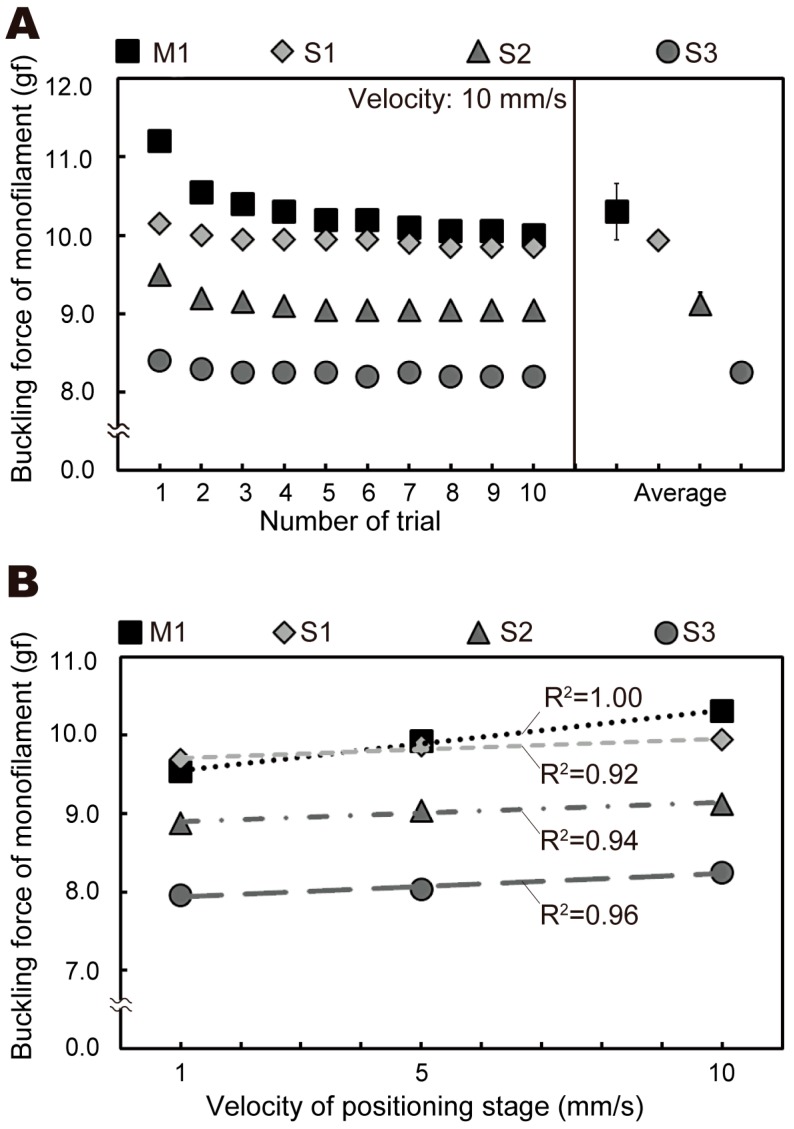
Experimental buckling force results for different monofilament types under automated operation using all four monofilaments, with one multi-use monofilament (denoted M1) and three single-use monofilaments (denoted S1, S2, and S3): (**A**) for each trial and (**B**) as a function of positioning stage velocity.

Finally, we compared the buckling force in a number of trials for other monofilaments, as shown in Figure 5. When the positioning stage velocity was 10 mm/s, the buckling force of the multi-use monofilament (M1) for the first compression was 11.2 gf, compared to 10.0 gf for the tenth trial. The buckling force of the single-use monofilament (S1) for the first and tenth trials was 10.1 gf and 9.8 gf, respectively. The average buckling force across all compressions was 10.3 ± 0.4 gf for M1, compared to 9.9 ± 0.1 gf for S1, 9.1 ± 0.1 gf for S2, and 8.2 ± 0.0 gf for S3 (see Figure 5A). Then, we compared the buckling force in the velocity of the positioning stage for all monofilaments, as shown in Figure 5B. The correlation coefficient (*R*^2^) between the buckling force of the multi-use monofilament (M1) and the number of trials was 1.00 (black markers), which indicates that the buckling force correlates closely with the number of compressions. The correlation coefficient (*R*^2^) between the buckling force of the three single-use monofilaments for S1, S2, and S3 and the number of trials was 0.92, 0.94, and 0.96, respectively. Hence, all the monofilaments correlated with the compression velocity.

## 4. Discussion

In the manual operation experiment (experiment 1), we measured the buckling force and compared the force values among 10 trials. The human hand motion with a monofilament differed among individuals, as in the previous study [21]. On average, although the buckling force monotonically decreased in 10 successive trials, the velocity of the hand motion and the contact angle did not show monotonic changes. Therefore, the force decline was considered to have not been caused by variations of the velocity of the hand motion and the contact angle.

In experiment 2, we replaced the human hand motion (manual operation) with an automated positioning stage (automated operation), to ensure the buckling force was not affected by variations of the velocity of the motion and the contact angle. In this case, the buckling force also decreased monotonically, as in the manual operation case. Although the human hand motion varied, the decline of the buckling force was shown to be related to the number of successive trials, rather than to the variations of hand motion. In our pilot study [21], we compared a new multi-use monofilament (with the same buckling force, i.e., 5.07/10 gf) with an old one, which was the same product as the multi-use monofilament. The buckling force of the monofilament decreased across the numerous test trials. The current experiments show that the decline of buckling force occurred not only in long-term use but also in short-term use. This problem was also addressed in a previous study in which Booth et al. [17] found that the maximum recovery in the buckling force in some monofilaments was achieved within 24 h.

In this study, the results indicate that the buckling force of the monofilament decreased by over 10% after 10 compressions. Weber’s law in psychophysics, which states that the relationship between a stimulus and its perception approximates a logarithmic function in human sensory systems, defines Weber’s fraction (K) of pressure on the skin to be approximately 10%. Hence, the reduction rate of the buckling force was considered to not be negligible for the SWMT. That is, the monofilament exhibits time-related deterioration characteristics from not only the long-term perspective but also the short-term perspective—a fact that should be considered by medical staff.

Furthermore, in experiment 2, in which the velocity and contact angle were automatically controlled, the buckling force of the monofilament was also affected by both the velocity of the hand motion and the contact angle. Hence, in the operation of the SWMT, the variability of the velocity of the human hand motion and the contact angles from the skin surface need to be considered.

In the automated operation experiment, with the different monofilament protocol (c), the buckling force of the monofilaments differed according to manufacturer. In particular, the multi-use monofilament gradually decreased between successive trials from the beginning tasks compared with the single-use monofilaments. The variability between monofilaments was more significant than the changes in force across the trials. Lambert et al. [24] have reported that the monofilaments can have 8% to 10% variability in their diameter due to variations in their manufacture. Furthermore, Bradman et al. [12] have reported that the buckling force of the monofilament can be affected by nearly 10% variability according to its diameter. Therefore, the diameter of the monofilament might influence the difference in the buckling force.

## 5. Conclusions

In this study, we found that the buckling force decreased across 10 successive trials, independent of the hand motion. Hence, the SWMT results varied not only due to the operator’s hand motion but also the effects of repeated trials. Therefore, the SWMT needs to take into consideration the number of compression trials. As the monofilaments showed time-related deterioration characteristics, we plan to develop a solution that minimizes the variability in the SWMT, and a new testing system [23] that uses tactile sensibility for diabetic peripheral neuropathy screening.
